# Improved Landmark Dynamic Prediction Model to Assess Cardiovascular Disease Risk in On-Treatment Blood Pressure Patients: A Simulation Study and Post Hoc Analysis on SPRINT Data

**DOI:** 10.1155/2020/2905167

**Published:** 2020-04-22

**Authors:** Mehrab Sayadi, Najaf Zare, Armin Attar, Seyyed Mohammad Taghi Ayatollahi

**Affiliations:** ^1^Cardiovascular Research Center, Shiraz University of Medical Sciences, Shiraz, Iran; ^2^Department of Biostatistics, School of Medicine, Shiraz University of Medical Sciences, Shiraz, Iran; ^3^Department of Biostatistics, Infertility Research Center, Shiraz University of Medical Sciences, Shiraz, Iran; ^4^Department of Cardiovascular Medicine, TAHA Clinical Trial Group, School of Medicine, Shiraz University of Medical Sciences, Shiraz, Iran

## Abstract

Landmark model (LM) is a dynamic prediction model that uses a longitudinal biomarker in time-to-event data to make prognosis prediction. This study was designed to improve this model and to apply it to assess the cardiovascular risk in on-treatment blood pressure patients. A frailty parameter was used in LM, landmark frailty model (LFM), to account the frailty of the patients and measure the correlation between different landmarks. The proposed model was compared with LM in different scenarios respecting data missing status, sample size (100, 200, and 400), landmarks (6, 12, 24, and 48), and failure percentage (30, 50, and 100%). Bias of parameter estimation and mean square error as well as deviance statistic between models were compared. Additionally, discrimination and calibration capability as the goodness of fit of the model were evaluated using dynamic concordance index (DCI), dynamic prediction error (DPE), and dynamic relative prediction error (DRPE). The proposed model was performed on blood pressure data, obtained from systolic blood pressure intervention trial (SPRINT), in order to calculate the cardiovascular risk. Dynpred, coxme, and coxphw packages in the R.3.4.3 software were used. It was proved that our proposed model, LFM, had a better performance than LM. Parameter estimation in LFM was closer to true values in comparison to that in LM. Deviance statistic showed that there was a statistically significant difference between the two models. In the landmark numbers 6, 12, and 24, the LFM had a higher DCI over time and the three landmarks showed better performance in discrimination. Both DPE and DRPE in LFM were lower in comparison to those in LM over time. It was indicated that LFM had better calibration in comparison to its peer. Moreover, real data showed that the structure of prognostic process was predicted better in LFM than in LM. Accordingly, it is recommended to use the LFM model for assessing cardiovascular risk due to its better performance.

## 1. Introduction

The risk prediction models (RPMs) are used as a diagnostic model to estimate the probability of an event occurrence in a disease or as a prognostic model to estimate probable consequences of a disease. Accurate prediction of a risk is essential in clinical research, and it is the patient's right to be informed about their disease progress [1]. Recently, RPMs are being used to help clinicians to make the best decision in diagnostic and therapeutic approaches, based on patient's demographics, test results, or disease characteristics [2]. Diagnostic models are usually used for risk classification in patients while prognostic models use time to assess disease progress [3, 4]. Nowadays, more prediction models have been used in cardiovascular diseases, such as diagnostic models for assessing the risk factors [3]. However, the cardiovascular risk assessment tools are static prediction models that use baseline predictors, but they still have some shortcomings, such as poor prediction [5], for instance, the inability to determine long-term survival of a heart attack patient with previous successful treatment or the inability to decrease the risk of cardiovascular event in a treated hypertensive patient. During the intervention, biomarkers are measured that are potentially informative in order to determine the treatment efficacy [6–9]. In this respect, the risk prediction using longitudinal biomarker is referred to as the dynamic prediction model (DPM), which was introduced by some researchers [10–13]. One DPM model is joint modeling (JM) [14–16], which requires correct determination of biomarker process distribution and event time, but this biomarker distribution is usually unclear. Moreover, its generalization to more than one marker leads to the production of ample computational complexity [17]. The landmark model (LM), another DPM, is used as an appropriate alternative to JM [9, 17–19]. The main advantage of LM is its simplicity, since it requires fewer assumptions compared to JM and might have much more power. In LM, the time is divided into different landmarks and then the simple Cox proportional hazards (PH) model is applied to each landmark for individuals who are still alive until time *t* [20, 21]. On the other hand, the biomarker value in each landmark time is considered as a fixed variable; hence, the prediction of risks becomes feasible. A landmark window should be considered to predict survival until time *sl* + *w*, which is called *t* horizon (thor). *w* is the length of time to predict patient survival as the prediction window.

By analyzing LM, the less frail patients are probably maintained dynamically during the landmark times. On the other hand, the estimated parameters in LM can be affected, if some patients do not follow the specific clinical visit schedule. Also, not considering the correlation between landmarks might affect the risk prediction. Bias in LM probably originates from these neglected issues. In order to improve LM, the frailty parameter was used to present a new model called the landmark frailty model (LFM). Finally, LFM was used to assess the cardiovascular risk in the on-treatment hypertensive patients. To reach this goal, a study with simulation data was designed, and the real blood pressure data which was obtained from systolic blood pressure intervention trial (SPRINT) was analyzed [22, 23].

The rest of this article is organized as follows.  Section 2 provides a brief description of the landmark approach as well as the proposed approach. Also, the setting of simulation studies, goodness of fit indices, and real data description are shown in Section 2. We conducted simulation studies to compare LFM with LM in Sections 3. In Section 4, we exhibited our approach with the SPRINT data followed by Section 5, which concludes and discusses simulation and real data.

## 2. Materials and Methods

### 2.1. Landmark Approach

Assuming that *T*_i_ and *C*_i_ are survival time (failure time) and censoring time, then *T*_i_^∗^ = min(*T*_i_, *C*_i_) make the observed time. *X* (.) represents the vector of covariates, which can be measured once at the beginning of the study. For example, age and gender are measured only at baseline and they are considered as fixed variables. *Y* (.) represents the longitudinal biomarker like systolic or diastolic blood pressure which can be measured for several time intervals. For risk assessment, the Cox (PH) model as the most famous model is defined as

(1) $$\begin{array}{c} h\left(t\right)=h_{0}\left(t\right)\exp\left(X\beta \right), \end{array}$$

where *h*(*t*) and *h*_0_(*t*) are hazard function at time *t* and baseline hazard, respectively. In LM, the time is divided into several landmark times including *s*_1_, *s*_2_, ⋯, *s*_*l*_. At landmark *l* (*l* = 1, 2, ⋯, *K*), subjects who are still at risk are considered for analysis and the remaining individuals will be omitted [9]. At each landmark, longitudinal biomarker value *Y*(*s*) is considered as a fixed variable. Then, a time period, a landmark window is considered to predict survival until time *sl* + *w* which is called *t* horizon (thor). *w* is the length of time to predict patient survival as prediction window, which is the so-called 3 or 5 years. The Cox PH model in equation (1) is reformulated and the conditional hazard function is estimated by

(2) $$\begin{array}{c} h\left(t\;|\;s,Y\left(S\right),X,w\right)=h_{l,0}\left(\left.t\right|s,w\right)\exp\left(Y\left(s_{l}\right)\alpha_{l}+\text{X}\beta_{l}\right),s\leq t\leq s+w. \end{array}$$

The model presented in equation (2) is defined as the simple or basic LM. It is used to fit a model to each landmark, and it estimates the specific landmark effect of a biomarker for predicting survival between *s*_*l*_ and *t*_thor_ where *h*_*l*,0_ is a different baseline hazard in each landmark. We assumed that a longitudinal biomarker, *Y*_*i*_(*t*_*ij*_), for subject *i* at the time of *j* was obtained from the mixed-effect model via the following formula:

(3) $$\begin{array}{c} Y_{i}\left(t_{ij}\right)=Z_{i}\left(t_{ij}\right)b_{gi}+X_{i}\left(t_{ij}\right)\beta +\varepsilon_{i}, \end{array}$$

where *Z*_*i*_ and  *X*_*i*_ denote the design vector for random and fixed effect and subscript *g* is 0 or 1.

According to equation (2), to consider the frailty of patients and the correlation between sequential measurements, LFM is defined as follows:

(4) $$\begin{array}{c} h\left(t\;|\;s,Y\left(s\right),X,u,w\right)=h_{l,0}\left(t\;|\;s,w\right)\exp\left(Y\left(s_{l}\right)\alpha_{l}+\text{X}\beta_{l}+u_{i}\right),s\leq t\leq s+w, \end{array}$$

where *u*_*i*_ indicates the frailty of patient *i*, which follows the multivariate normal distribution with mean 0 and covariance matrix *Σ*(*θ*). The survival prediction model is related to cumulative hazard function by

(5) $$\begin{array}{c} S\left(t+w\;|\;s,Y\left(s\right),X,u,w\right)=\exp\left(-\text{H}\left(t\;|\;s,Y\left(s\right.\right),X,u,w\right),s\leq t\leq s+w. \end{array}$$

To estimate the parameters, Cox partial likelihood is modified via the following integrated (over landmarks) partial log-likelihood (IPL) [24].

(6) $$\begin{array}{c} \text{IPL}\left(\alpha ,\beta ,u\right)=\overset{n}{\underset{i=1}{\sum }}d_{i}\underset{l}{\sum }\ln\left(\frac{\exp\left(Y_{i}\left(s_{l}\right)\alpha_{l}+X_{i}\beta_{l}+u_{i}\right)}{\sum_{j\epsilon R\left(s_{l}\right)}\exp\left(Y_{j}\left(s_{l}\right)\alpha_{l}+X_{j}\beta_{l}+u_{j}\right)}\right). \end{array}$$

In this formula, *d*_*i*_ indicates the risk set, *d*_*i*_ = 1 if subject *i* remains until time *s* at landmark *l*. Otherwise, *d*_*i*_ is assumed 0. *R*(*s*_*l*_) denotes the risk set at time *s* at landmark *l*. We used the integrated partial likelihood (IPL^∗^) by integrating the random effects [25].

(7) $$\begin{array}{c} {\text{IPL}}^{*}\left(\alpha ,\beta ,\theta \right)=\int \text{IPL}\left(\alpha ,\beta ,u\right)\;f\left(u\right)du. \end{array}$$

By maximizing the IPL^∗^, maximum likelihood estimators (MLE) for the parameters are provided. In addition, the coxme function in coxme package can only provide an ML estimate. With respect to the complexity of IPL^∗^ calculation, the coxme package uses the Laplace approximation technique. More details are described elsewhere [25, 26].

We can also perform a model for all landmarks by stacking data set defined as super LFM, which considers parameters as if they depended on the time in a smooth fashion. It is formulated as

(8) $$\begin{array}{c} h\left(t\;|\;s,Y\left(s\right),X,u,w\right)=h_{0}\left(t\;|\;s,w\right)\exp\left(Y\left(s\right)\alpha \left(s\right)+X\beta_{s}+u_{i}\right), \end{array}$$

where

(9) $$\begin{array}{c} \alpha \left(s\right)=\overset{m_{b}}{\underset{j=1}{\sum }}\gamma_{j}f_{j}\left(s\right). \end{array}$$

Dynpred, coxme, and coxphw packages in the R.3.4.3 software were used for data analysis.

### 2.2. Simulation Study Setting

To assess the application of the models in different aspects, we set up several scenarios with different specificities in terms of sample size (*n* = 100, 200, and 400), number of landmarks (6, 12, 24, and 48), failure rate (30, 50, and 100%), and complete/missing data. In order to perform these models, a dichotomous variable with binomial distribution like treatment effect and continuous covariate with normal distribution like age (*X*_1_ and *X*_2_, respectively) were considered. The regression coefficient, *β*_1_ and *β*_2_, was set at 0.5 and 1.5 as true values, respectively, for *X*_1_ and *X*_2_. In equation (3), we assume that *b*_*g*_ has a bivariate normal distribution for random intercept and slope with a mean of 0 and covariance matrix of *δ*_11_ = 2, *δ*_12_ = 0.2, *δ*_22_ = 1. We also assumed that the individual error term (*ε*_*i*_) follows a normal distribution with a mean of 0 and variance of 1. Moreover, it is assumed that continuous variable *Y* was measured for 10 times for each individual sequentially. The time *T* was generated from Weibull distribution [27] as shown in the following:

(10) $$\begin{array}{c} T={\left[\frac{1}{k}\ln\left(1+\frac{\left(1+\lambda \right)\left(-\ln\left(\nu \right)\right)}{\gamma \exp\left(X\beta \right)\varphi \lambda }\right)\right]}^{1/\left(1+\lambda \right)}. \end{array}$$

In this equation, *k* = 1.1, *γ* = 0.4, *λ* = 0.01, *φ* = 0.75, and *v* has a uniform (0, 1) distribution. Moreover, we performed the simple Cox model that just included the baseline data in three different sample sizes and three different failure percentages.

### 2.3. Goodness of Fit (GOF) and Prediction Ability Indices

There are several indices to assess the goodness of fit (GOF) and prediction ability in DPM. We used the standard error, bias of parameter estimation, and the mean square error (MSE), which were obtained from 300 simulation data. To compare LFM with LM, log-likelihood as well as deviance statistic was used. The latter is compared with mixture chi-square value (1.92) that was obtained from  (1/2)(*χ*_0_^2^ + *χ*_1_^2^). Akaike information criterion (AIC) was used as if smaller AIC implies a better fit. Moreover, the dynamic concordance index (DCI), dynamic prediction error (DPE), and dynamic relative prediction error (DRPE) were used to measure the discrimination and calibration ability. DPE was obtained from the Brier error score formula:

(11) $$\begin{array}{c} \text{Brier error}=\frac{1}{n}\overset{n\;}{\underset{i=1}{\sum }}\left(d_{i}\left(t\right)-\hat{S_{i}}{\left(t\right)}^{2}\right). \end{array}$$

In fact, the Brier score measures the average discrepancies between true event status and predictive values of survival at time *t*. Low Brier score of a model indicates the better predictive performance of that model. In this formula, *d*_*i*_ as the actual observation for subject *i* at time *t* is an event status, which could be either 1 or 0 (the occurrence or nonoccurrence of an event, respectively).

The predicted survival $\left(\hat{S_{i}}\right)$ is estimated by model LM or LFM [28]. DRPE was calculated from

(12) $$\begin{array}{c} \text{Relative Prediction Error}=\left(1-\frac{\text{null model error}}{\text{curent model error}}\;\right). \end{array}$$

In equation (12), errors are obtained from equation (11) and the null model is a model without any covariates, such as Kaplan-Meier estimate, and the current model is LM or LFM.

### 2.4. Real Data

We used a part of the systolic blood pressure intervention trial (SPRINT) study [29] (National Heart, Lung, and Blood Institute (NHLBI), funded by the National Institutes of Health; ClinicalTrials.gov number NCT01206062) upon a request ID of 4612. In the main study of SPRINT, methods were reported in detail [30]. In summary, in that randomized controlled trial study, 9361 nondiabetic participants with systolic blood pressure (SBP) of equal or more than130 mmHg were allocated to an intensive treatment (target SBP < 120 mmHg) and standard treatment (target SBP < 140 mmHg) groups. Baseline data, lab data, and repeated measurement of SBP for 21 times in 5 years were collected. Heart failure, stroke, myocardial infarction, other acute coronary syndromes, and death from cardiovascular causes were regarded as cardiovascular events. Hence, we designed a case-cohort study from this data, which included Framingham risk factors of age, gender, total cholesterol (TCH) level, high-density lipoprotein cholesterol (HDL) level, and SBP. In our model, treatment effect was added to the abovementioned data. We considered 10 measurements of SBP (baseline, 6, 12, 18, 24, 30, 36, 42, 48, and 54 months). The aim was to determine the dynamic effect of blood pressure on cardiovascular disease risk by comparing LFM with LM. On the other hand, these two models were compared with the simple Cox model by considering only baseline blood pressure data. To compare LFM with LM, we used AIC and deviance criteria. And the deviance criteria were tested using mixture chi-square.

## 3. Results of Simulation

Simple Cox model results are summarized in Table 1, and results of LM and LFM are summarized in Tables 2–4

### 3.1. Landmark Models vs. Simple Cox Model

Both LFM and LM had a better relative performance in comparison to the simple Cox model. As can be seen in all scenarios, bias and MSE of bias were lower in LMs; however, this difference decreased as the sample size increased from 100 to 200-400. Nevertheless, bias did not have basic changes in failure rate.

### 3.2. Comparing LFM vs. LM

The performance of the models was evaluated based on their ability to estimate the true value of the parameters and their ability to classify and predict the actual survival.

#### 3.2.1. Ability to Estimate the True Value of the Parameters

Mean of parameter estimation and its SE, bias, and MSE for failure rate (30%) are shown in Table 2. In total, bias and MSE were lower in LFM in comparison with LM. According to deviance and AIC indices, using 12 landmarks and sample size of 100, there was no statistically significant difference between LFM and LM in both data sets. Deviance was 1.76 in complete data and 1.11 in incomplete data. Deviance index showed that there was a statistically significant difference between the two models at the sample size of 200 and 400 in the complete data. At the sample size of 200, AICs were 1515 and 3636 in LFM and LM, respectively, while at the sample of 400 they were 1521 and 3648.

In these cases, bias and MSE of bias for the two parameters were slightly lower in LFM. In the incomplete data, according to deviance index, there was no significant difference between the two models in all sample sizes, while in the complete data (200-400) statistically significant difference was observed (in both cases, deviance was greater than mixture chi-square and AICs were lower in LFM). In all scenarios with 24 and 40 landmarks, based on deviance and AIC indices, LFM fitted better in comparison to LM. In these cases, the mean estimation of two parameters in the LFM was closer to the true value. This result was more pronounced in the continuous variable. According to the results of failure rate of 50% and 100% summarized in Tables 3 and 4, the superiority of LFM over LM was higher, especially in the model with 12 landmarks.

#### 3.2.2. Ability to Classify and Predict the Actual Survival

**Figure 1:**
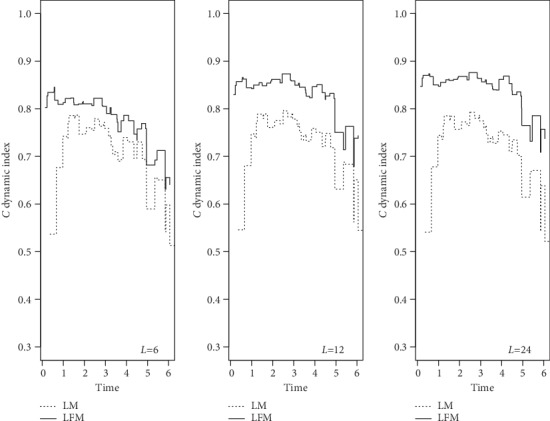
Simulated *C* dynamic index for landmark model (LM) and landmark frailty model (LFM): landmarks = 6, 12, and 24; sample size = 200; and failure rate = 50%. The higher values of *C* index indicate more discrimination ability.

**Figure 2:**
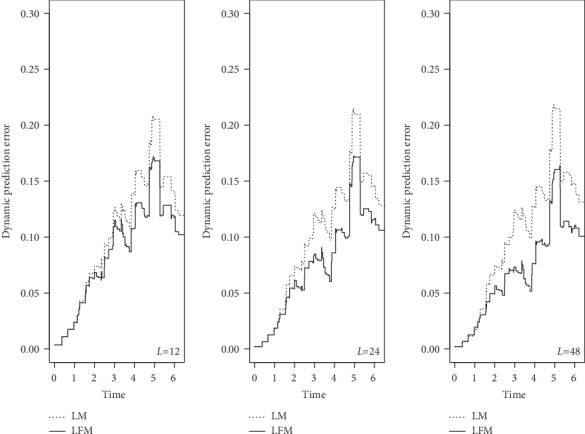
Simulated dynamic prediction error for landmark model (LM) and landmark frailty model (LFM): landmarks = 6, 12, and 24; sample size = 200; and failure rate = 50%. The lower values of prediction error indicate more calibration.

**Figure 3:**
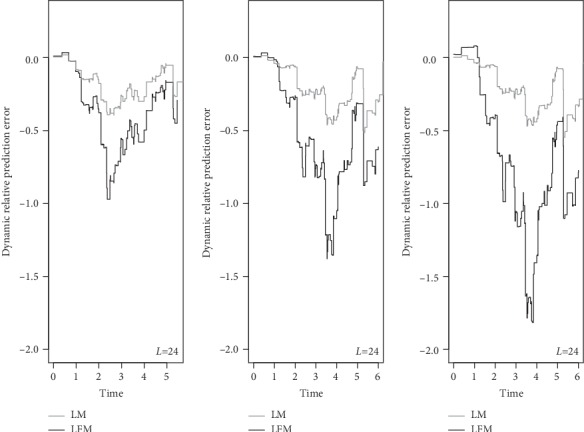
Simulated dynamic relative prediction error for landmark model (LM) and landmark frailty model (LFM): landmarks = 6, 12, and 24; sample size = 200; and failure rate = 50%. The figures show which model has been able to reduce the error more.

We used CDI to assess the discrimination ability of the two models that were run with fixed sample size and failure rate in 3 different landmarks (6, 12, and 24), where CDI value is greater than 0.5, indicating that the model had discrimination ability. As illustrated in Figure 1, LFM had better performance. This advantage was more evident by increasing the number of landmarks from 6 to 12-24. On the other hand, the more area under the curve indicated the more accurate model. DPE and DRPE for calibration ability were plotted in Figures 2 and 3. The error rates and relative error rates in LFM were much lower than those in LM, and this became more prominent by increasing landmark numbers.

## 4. Results of Real Data

**Figure 4:**
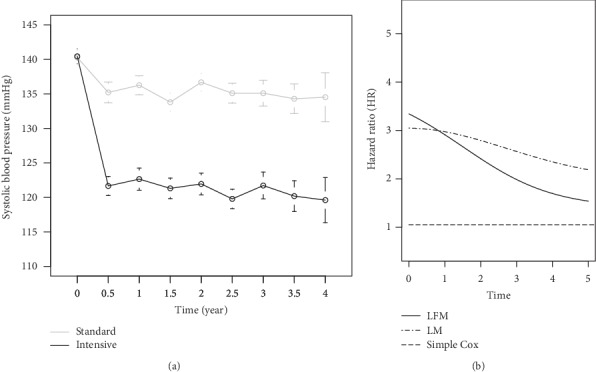
(a) Systolic blood pressure (SBP) in the two treatment groups over time. Target of SBP in the intensive group and the standard group was less than 120 mmHg and 140 mmHg, respectively. (b) Hazard ratio prediction of systolic blood pressure over time adjusted for treatment effect and other covariates. It is shown that as blood pressure decreases over time due to treatment effect, the risk also decreases. This reduction is more in LFM. Also, the hazard ratio in the simple Cox model (included just baseline SBP) is fixed over time. AIC = 5437 and 6385 in LFM and LM, respectively, also deviance = 559.7 (*p* < 0.001).

**Figure 5:**
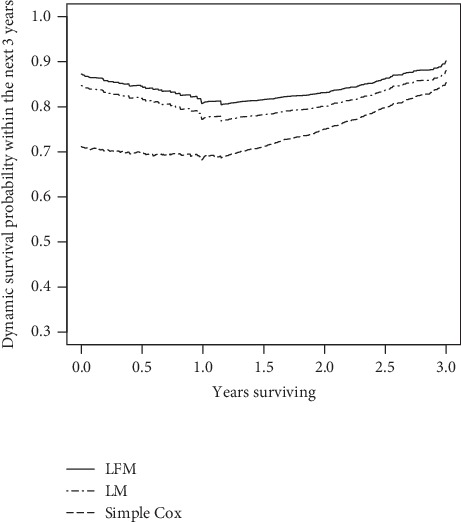
Dynamic prediction of survival within window (*w* = 3) by adjusting the covariates in LFM, LM, and simple Cox model.

Results of real data are summarized in Table 5 and Figure 4. The adjusted hazard ratio of variables and its confidence interval (CI) are provided. Furthermore, AIC and deviance index were extracted to assess the models. While in both LFM and LM the SBP was highly significant, it had no significant impact on cardiovascular events in simple Cox (*p* value = 0.258). However, LFM fitted better since it had an AIC equal to 5437 while it was 6385 in LM. Also, the deviance between two models was 559.7 (*p* < 0.001). After adjusting the treatment effect as well as baseline risk factor effect (Figure 4), it was shown that while SBP was decreasing over time, the hazard ratio (HR) was decreasing in line with SBP in both models. However, it is noteworthy that this reduction was more in the LFM. On the contrary, HR was constant over time in the Cox model. As the blood pressure decreases, the 3-year survival prediction increases. LFM predicts higher survival than LM and the simple Cox model (Figure 5).

## 5. Discussion

### 5.1. Discussion of Simulation Data

DPM includes time-dependent marker information during follow-up in order to improve personal survival prediction probabilities. At any follow-up, time-updated marker value can be used to generate a dynamic prediction [10–12]. These models are essential to identify high-risk individuals and timely clinical decision-making. Recently, LM as DPM was extensively investigated by researchers [9, 24]. Some of them used LM in different aspects of survival data such as competing risk and cure data [14, 20]. However, they paid little attention to individuals' frailty and regularity of visits as well as correlation between different landmarks. On the other hand, LM can be affected by the way how landmarks are selected and number of landmarks. Ignoring these issues might lead to an estimation error. Hence, we proposed a modified LM which used the frail parameter as LFM. Indeed, in LM, individuals who experienced the intended event or being censored at a defined landmark time are considered in data analysis. Frailty plays a critical role in those who are retained and repeated dynamically in sequential landmarks due to their low frailty. However, in our proposed model, considering the frailty of patients was included in the analysis; hence, we were able to overcome the abovementioned problems. Simulations showed that both LFM and LM had a relative advantage over the simple Cox model. This was confirmed by various criteria such as bias and MSE. Bias and MSE in dichotomous and continuous variables were higher in the simple Cox model, which is in line with other studies [20, 31]. In the simple Cox model, as the sample size increased, the estimation error slightly decreased and the estimates were closer to the true values. But the simple Cox model was still behind the LMs. LFM and LM were compared regarding different sample sizes, different number of landmarks, different failure rate, and diverse data structure. Generally, the superiority of the LFM over LM was confirmed in the present study. This conformation was very clear in a large sample size and higher landmark number in both complete and incomplete data.

To the best of our knowledge, this is the first study to investigate the effect of number of landmarks on accuracy of the results. Wright et al. [31] performed LM with 20 landmarks, and others have empirically found that 20 to 100 landmarks are appropriate [24]. However, in the case of large sample size, data become too large, and it took too much time to run the programs. There was no significant difference between LM and LFM in small sample size and low number of landmarks, which was the result of checking the deviance and AIC indices between the two models. Both models performed better by increasing failure rate. Although no statistical comparison was made, in each case, LFM was more appropriately fitted with fewer estimation errors. In most times, the discrimination ability of LFM was more than LM since DCI was more than 70% in LFM while this index was lower in LM. The difference between DCIs increased by the implementation of increased number of landmarks. Also, evaluation of collaboration ability (DPE and DRPE) showed that LMF had a better performance than LM.

### 5.2. Discussion of SPRINT Data

Hypertension is not only recognized as a major cardiovascular risk factor but also has a significant impact on the occurrence of events followed by therapeutic interventions [32]. In this study, we showed that considering hypertension as a dynamic risk factor had a basic role for obtaining real estimation of successful treatment in cardiovascular diseases. Hence, it should be considered dynamically during the course of treatment and not only at the time of admission as a primary measure that it was emphasized by other similar studies [9, 33]. SPRINT data confirmed the simulation results, which contained repeated measurements of SBP as a single longitudinal biomarker. As mentioned in the previous section, more than one biomarker could be used in LMs. By only using the baseline blood pressure data (simple Cox model), the role of SBP was hindered due to dominancy of treatment effect; hence, it was not recognized as a cardiovascular risk factor. This result is in line with the results of our previous study [22] and the same result was obtained from the study carried out by Group S.R. [29]. In both landmark models, as SBP decreased after treatment, the risky effect of SBP was also reduced. While HR in the simple Cox model is close to 1 and constant over time, HR in the two landmark models was close to 3 at the beginning of the study and then it relatively decreased by decreasing blood pressure over time, although the intensity of the significant reduction was higher in our proposed model. On the other hand, in the simple Cox model, the effect of intensive treatment was 38% (1-1/0.720) in comparison with standard treatment, while in LFM and LM it was 110% and 107%, respectively. This means that the protective effect of intensive treatment is highly exhibited in our model in comparison with simple Cox and LM. Thereby, predictability of the 3-year dynamic survival is higher in LFM. Other studies that worked on blood pressure have considered landmarks separately while we used a model that landmarks were considered continuously [21, 33, 34]. This study showed that landmark models can be used to help clinicians to make better decision for diagnosis and treatment. Landmark models, especially our proposed model, are useful for risk assessment, when the data is not complete or regular, similar to our data.

## 6. Conclusion

In this study, we provided a modified LM, which considered the frailty of the patients as well as the correlation between the landmarks. Our approach can be fitted better in the sense that it has a better GOF, improved real data analysis, and more optimized cardiovascular risk assessment.

## Figures and Tables

**Table 1:** Simple Cox model results in different simulations.

*n*	Estimate	Failure = 30%		Failure = 50%		Failure = 100%	
		*β*_1_ = 0.5	*β*_2_ = 1.5	*β*_1_ = 0.5	*β*_2_ = 1.5	*β*_1_ = 0.5	*β*_2_ = 1.5
100	Mean	0.384	1.290	0.384	1.286	0.401	1.103
	SE	0.513	0.368	0.543	0.392	0.318	0.226
	Bias	-0.116	-0.210	-0.115	-0.213	-0.099	-0.397
	MSE	0.677	0.464	0.377	0.304	0.158	0.128

200	Mean	0.402	1.327	0.417	1.298	0.420	1.285
	SE	0.750	0.549	0.246	0.174	0.252	0.180
	Bias	-0.098	-0.173	-0.082	-0.202	-0.08	-0.215
	MSE	0.720	0.692	0.065	0.084	0.080	0.097

400	Mean	0.405	1.287	0.438	1.308	0.431	1.300
	SE	0.320	0.226	0.252	0.180	0.175	0.123
	Bias	-0.095	-0.213	-0.062	-0.191	-0.069	-0.200
	MSE	0.106	0.326	0.368	0.125	0.036	0.059

**Table 2:** LM and LFM results in different scenarios when failure rate is 30%.

*L*	*n*	Estimate	Data without missing							Data with missing						
			LM			LFM			*D*	LM			LFM			*D*
			*β*_1_ = 0.5	*β*_2_ = 1.5	AIC	*β*_1_ = 0.5	*β*_2_ = 1.5	AIC		*β*_1_ = 0.5	*β*_2_ = 1.5	AIC	*β*_1_ = 0.5	*β*_2_ = 1.5	AIC	
12	100	Mean	0.418	1.406	619	0.422	1.429	614	1.76	0.408	1.421	244	0.435	1.422	241	1.11
		SE	0.224	0.182		0.219	0.180			0.339	0.274		0.329	0.280		
		Bias	-0.081	-0.093		-0.077	-0.070			-0.091	-0.078		-0.065	-0.078		
		MSE	0.201	0.138		0.210	0.140			0.315	0.680		0.270	0.177		
	200	Mean	0.459	1.405	1521	0.465	1.420	1515	2.39	0.458	1.402	624	0.466	1.422	621	1.01
		SE	0.149	0.125		0.150	0.123			0.217	0.180		0.220	0.183		
		Bias	-0.040	-0.095		-0.034	-0.079			-0.042	-0.097		-0.033	-0.077		
		MSE	0.087	-0.064		0.086	0.063			0.107	0.328		0.110	0.323		
	400	Mean	0.463	1.400	3646	0.468	1.415	3636	4.05	0.458	1.411	1537	0.464	1.430	1532	1.79
		SE	0.103	0.086		0.104	0.085			0.147	0.122		0.150	0.124		
		Bias	-0.036	-0.100		-0.032	-0.085			-0.041	-0.089		-0.035	-0.070		
		MSE	0.039	0.039		0.040	0.038			0.053	-0.049		0.054	0.048		

24	100	Mean	0.419	1.405	1233	0.449	1.509	1167	36.18	0.431	1.421	481	0.504	1.536	433	25.41
		SE	0.171	0.144		0.155	0.128			0.232	0.194		0.285	0.203		
		Bias	-0.081	-0.094		-0.051	0.009			-0.069	-0.078		0.004	0.036		
		MSE	0.199	0.139		0.234	0.125			0.265	0.174		0.414	0.306		
	200	Mean	0.460	1.400	3048	0.495	1.500	2912	71.26	0.461	1.399	1246	0.539	1.590	1143	52.59
		SE	0.105	0.024		0.115	0.020			0.152	0.127		0.181	0.152		
		Bias	-0.039	-0.100		-0.005	0.000			-0.038	-0.101		0.038	0.059		
		MSE	0.090	0.066		0.098	0.064			0.110	0.086		0.096	0.120		
	400	Mean	0.463	1.401	7872	0.498	1.500	7011	144.43	0.456	1.412	3064	0.513	1.579	2854	107.85
		SE	0.079	0.060		0.072	0.020			0.104	0.086		0.122	0.102		
		Bias	-0.037	-0.099		-0.002	0.000			-0.044	-0.088		0.013	0.079		
		MSE	0.039	0.039		0.040	0.036			0.052	0.048		0.069	0.074		

40	100	Mean	0.421	1.409	1808	0.462	1.545	1664	90.28	0.431	1.420	704	0.534	1.558	591	69.02
		SE	0.127	0.106		0.145	0.123			0.191	0.159		0.251	0.213		
		Bias	-0.078	-0.090		-0.038	0.045			-0.069	-0.079		0.034	0.058		
		MSE	0.200	0.137		0.246	0.160			0.261	-0.174		0.469	0.395		
	200	Mean	0.460	1.399	4478	0.504	1.544	4178	185.16	0.460	1.401	1829	0.556	1.564	1596	142.63
		SE	0.097	0.083		0.086	0.072			0.125	0.105		0.155	0.132		
		Bias	-0.040	-0.101		0.004	0.044			-0.040	-0.099		0.056	0.064		
		MSE	0.105	0.074		0.091	0.064			-0.111	0.080		0.168	0.161		
	400	Mean	0.465	1.403	10705	0.513	1.541	10106	371.92	0.458	1.413	4501	0.538	1.563	4022	291.24
		SE	0.060	0.050		0.067	0.050			0.086	0.072		0.105	0.088		
		Bias	-0.035	-0.096		0.013	0.041			-0.042	-0.068		0.038	0.063		
		MSE	0.048	0.040		0.040	0.040			0.054	0.048		0.078	0.099		

*L* = landmarks; *n* = sample size; LM = landmark model; LFM = landmark frailty model; AIC = Akaike information criterion; *D* = deviance; SE = standard error; MSE = mean square error.

**Table 3:** LM and LFM results in different scenarios when failure rate is 50%.

*L*	*n*	Estimate	Data without missing							Data with missing						
			LM			LFM			*D*	LM			LFM			*D*
			*β*_1_ = 0.5	*β*_2_ = 1.5	AIC	*β*_1_ = 0.5	*β*_2_ = 1.5	AIC		*β*_1_ = 0.5	*β*_2_ = 1.5	AIC	*β*_1_ = 0.5	*β*_2_ = 1.5	AIC	
12	100	Mean	0.438	1.376	1053	0.445	1.402	1045	2.91	0.443	1.407	416	0.453	1.439	412	1.10
		SE	0.165	0.138		0.170	0.141			0.246	0.204		0.223	0.210		
		Bias	-0.062	-0.123		-0.055	-0.097			-0.057	-0.093		-0.046	-0.061		
		MSE	0.097	0.084		0.100	0.082			0.130	0.100		0.137	0.106		
	200	Mean	0.462	1.397	2562	0.467	1.414	2552	3.52	0.467	1.401	1047	0.474	1.425	1043	1.60
		SE	0.113	0.095		0.115	0.097			0.165	0.141		0.167	0.139		
		Bias	-0.038	-0.103		-0.032	-0.085			-0.033	-0.098		-0.025	-0.074		
		MSE	0.050	0.045		0.051	0.043			0.065	0.053		0.065	0.051		
	400	Mean	0.456	1.400	6092	0.462	1.416	6076	6.22	0.457	1.408	2581	0.465	1.429	2573	2.63
		SE	0.080	0.066		0.080	0.066			0.113	0.094		0.115	0.095		
		Bias	-0.044	-0.100		-0.038	-0.084			-0.043	-0.092		-0.035	-0.071		
		MSE	0.022	0.027		0.022	0.024			0.029	0.032		0.029	0.029		

24	100	Mean	0.436	1.374	213	0.473	1.485	2006	49.95	0.438	1.403	826	0.506	1.541	751	35.88
		SE	0.117	0.098		0.130	0.101			0.173	0.145		0.212	0.179		
		Bias	-0.064	-0.126		-0.027	-0.015			-0.062	-0.097		0.006	0.041		
		MSE	0.096	0.088		0.112	0.084			0.129	0.101		0.200	0.192		
	200	Mean	0.462	1.396	5121	0.496	1.500	4923	97.54	0.465	1.400	2092	0.543	1.514	1940	71.14
		SE	0.080	0.067		0.088	0.070			0.117	0.098		0.139	0.118		
		Bias	-0.038	-0.104		-0.003	0.000			-0.034	-0.100		0.043	0.014		
		MSE	0.050	0.045		0.057	0.044			0.065	0.052		0.092	0.080		
	400	Mean	0.456	1.400	12179	0.490	1.500	11794	191.85	0.457	1.409	5155	0.522	1.554	4848	145.23
		SE	0.065	0.047		0.061	0.051			0.080	0.066		0.094	0.078		
		Bias	-0.044	-0.100		-0.010	0.000			-0.043	-0.091		0.022	0.054		
		MSE	0.022	0.026		0.024	0.021			0.028	0.032		0.041	0.049		

40	100	Mean	0.438	1.377	3086	0.486	1.520	2880	124.04	0.438	1.403	1211	0.535	1.568	1045	96.44
		SE	0.096	0.080		0.110	0.093			0.143	0.135		0.185	0.158		
		Bias	-0.062	-0.123		-0.014	0.020			-0.062	-0.097		0.035	0.068		
		MSE	0.095	0.083		0.121	0.095			0.128	0.101		0.237	0.250		
	200	Mean	0.452	1.407	7520	0.500	1.525	7107	245.49	0.455	1.413	3100	0.552	1.561	2762	195.96
		SE	0.077	0.055		0.074	0.024			0.096	0.080		0.120	0.103		
		Bias	-0.048	-0.092		0.000	0.025			-0.045	-0.087		0.052	0.061		
		MSE	0.043	0.040		0.051	0.045			0.053	0.053		0.086	0.128		
	400	Mean	0.457	1.402	17912	0.503	1.539	17089	488.90	0.458	1.409	5150	0.509	1.558	4841	143.24
		SE	0.046	0.038		0.051	0.040			0.066	0.055		0.045	0.038		
		Bias	-0.043	-0.098		0.003	0.039			-0.042	-0.091		0.009	0.058		
		MSE	0.022	0.026		0.025	0.025			0.029	0.031		0.049	0.028		

*L* = landmarks; *n* = sample size; LM = landmark model; LFM = landmark frailty model; AIC = Akaike information criterion; *D* = deviance; SE = standard error; MSE = mean square error.

**Table 4:** LM and LFM results in different scenarios when failure rate is 100%.

*L*	*n*	Estimate	Data without missing							Data with missing						
			LM			LFM			*D*	LM			LFM			*D*
			*β*_1_ = 0.5	*β*_2_ = 1.5	AIC	*β*_1_ = 0.5	*β*_2_ = 1.5	AIC		*β*_1_ = 0.5	*β*_2_ = 1.5	AIC	*β*_1_ = 0.5	*β*_2_ = 1.5	AIC	
12	100	Mean	0.390	1.197	1785	0.395	1.216	1776	3.39	0.383	1.205	707	0.392	1.232	703	1.36
		SE	0.099	0.083		0.102	0.085			0.147	0.123		0.150	0.120		
		Bias	-0.110	-0.303		-0.105	-0.284			-0.117	-0.295		-0.108	-0.268		
		MSE	0.045	0.035		0.046	0.034			0.064	0.046		0.064	0.045		
	200	Mean	0.474	1.394	5147	0.480	1.411	5135	5.67	0.480	1.390	2114	0.488	1.421	2109	2.19
		SE	0.081	0.066		0.080	0.068			0.115	0.096		0.118	0.099		
		Bias	-0.026	-0.106		-0.020	-0.089			-0.020	-0.110		-0.012	-0.079		
		MSE	0.023	0.027		0.023	0.025			0.029	0.031		0.029	0.028		
	400	Mean	0.464	1.393	12176	0.468	1.408	12155	11.02	0.465	1.397	5156	0.472	1.420	5145	4.88
		SE	0.055	0.046		0.057	0.047			0.080	0.067		0.082	0.068		
		Bias	-0.036	-0.107		-0.032	-0.091			-0.036	-0.103		-0.028	-0.080		
		MSE	0.012	0.018		0.011	0.015			0.015	0.021		0.015	0.017		

24	100	Mean	0.388	1.195	3567	0.410	1.275	3455	53.91	0.382	1.204	1408	0.432	1.382	1324	37.27
		SE	0.070	.058		0.077	0.065			0.104	0.087		0.124	0.105		
		Bias	-0.112	-0.305		-0.090	-0.225			-0.117	-0.295		-0.067	-0.118		
		MSE	0.045	0.039		0.054	0.036			0.062	0.046		0.088	0.088		
	200	Mean	0.475	1.395	10297	0.500	1.478	10071	107.83	0.480	1.398	4228	0.546	1.477	4056	74.70
		SE	0.056	0.048		0.061	0.051			0.082	0.068		0.095	0.080		
		Bias	-0.025	-0.105		0.000	-0.022			-0.020	-0.102		0.046	-0.023		
		MSE	0.022	0.028		0.027	0.025			0.029	0.031		0.044	0.043		
	400	Mean	0.464	1.391	24354	0.486	1.465	23904	215.03	0.464	1.397	10313	0.514	1.537	9964	154.33
		SE	0.039	0.032		0.042	0.035			0.056	0.047		0.065	0.054		
		Bias	-0.036	-0.109		-0.014	-0.035			-0.036	-0.103		0.014	0.037		
		MSE	0.012	0.018		0.012	0.011			0.015	0.021		0.022	0.020		

40	100	Mean	0.388	1.196	5239	0.419	1.311	5006	132.35	0.382	1.205	2065	0.458	1.472	1874	101.58
		SE	0.057	0.048		0.065	0.056			0.085	0.072		0.109	0.094		
		Bias	-0.112	-0.304		-0.080	-0.189			-0.118	-0.295		-0.042	-0.027		
		MSE	0.045	0.036		0.058	0.044			0.064	0.047		0.112	0.133		
	200	Mean	0.476	1.396	15141	0.522	1.521	14643	276.26	0.482	1.401	6211	0.581	1.575	5799	214.15
		SE	0.046	0.039		0.052	0.044			0.067	0.056		0.083	0.071		
		Bias	-0.023	-0.103		0.022	0.021			-0.018	-0.099		0.081	0.075		
		MSE	0.023	0.027		0.030	0.028			0.029	0.030		.058	0.077		
	400	Mean	0.464	1.393	35826	0.501	1.507	34828	555.80	0.465	1.397	15169	0.541	1.557	14730	430.87
		SE	0.032	0.027		0.036	0.030			0.046	0.038		0.036	0.068		
		Bias	-0.036	-0.107		0.001	0.007			-0.035	-0.103		0.041	0.057		
		MSE	0.012	0.018		0.013	0.011			0.014	0.020		0.028	0.043		

*L* = landmarks; *n* = sample size; LM = landmark model; LFM = landmark frailty model; AIC = Akaike information criterion; *D* = deviance; SE = standard error; MSE = mean square error.

**Table 5:** Static and dynamic effect of SBP on cardiovascular event.

Variables	Simple Cox		LM		LFM	
	HR	*p* value	HR	*p* value	HR	*p* value
Age _at enrolment day, yr_	1.051	<0.001	1.018	<0.001	1.022	<0.001
Gender (female)	0.691	<0.001	1.019	0.164	0.843	0.291
TCH (mmol/L)	1.001	<0.001	1.001	0.163	1.000	0.582
HDL-C (mmol/L)	0.986	<0.001	0.991	0.001	0.987	0.023
Current smoker	1.820	<0.001	1.178	0.040	1.271	0.045
Treatment, intensive	0.720	<0.001	0.482	<0.001	0.475	<0.001
SBP (mmHg)	1.001	0.258	1.083	<0.001	1.109	<0.001
SBP^∗^time	—	—	0.996	<0.001	0.839	<0.001
SBP^∗^time^2^	—	—	0.966	<0.001	0.895	<0.001

LM = landmark model; LFM = landmark frailty model; HR = hazard ratio; TCH = total cholesterol; HDL-C = high-density lipoprotein; SBP = systolic blood pressure.

## Data Availability

The data used to support the findings of this study were supplied by [National Heart, Lung, and Blood Institute (NHLBI),] under license and so cannot be made freely available. Requests for access to these data should be made to {National Heart, Lung, and Blood Institute (NHLBI), Funded by the National Institutes of Health; ClinicalTrials.gov number, NCT01206062}. We have a request ID of 4612.
